# Supplementary material for: Tunable Composition of Dynamic Non-Viral Vectors over the DNA Polyplex Formation and Nucleic Acid Transfection
Source: Polymers (Basel). 2019 Aug 6;11(8):1313. doi: 10.3390/polym11081313 (PMC6724009; doi:10.3390/polym11081313)
Supplement: Supplementary file 1 [file polymers-11-01313-s001.pdf]

Tunable composition of dynamic non-viral vectors over the DNA polyplex formation mechanism and nucleic acid transfection

Lilia Clima*, Bogdan Florin Craciun, Gabriela Gavril and Mariana Pinteala*

Synthesis of PEGylated squalene

PEGylated squalene was synthesized in four steps as reported in previous papers [1-3]. Briefly, squalene was functionalized to 1,1′,2-tris-nor-squalene aldehyde in three steps. In the first step 2-hydroxy-3-bromosqualene was synthetized from squalene with *N*-bromosuccinimide in tetrahydrofuran at 0 °C for 90 minutes, then, 2,3-oxidosqualene was obtained from 2-hydroxy-3-bromosqualene with potassium carbonate in methanol at room temperature (25 °C) for 2 hours. In the third step, 1,1′,2-tris-nor-squalene aldehyde was obtained by reducing the epoxide with periodic acid in water-dioxane solution at room temperature (25 °C) for 2 hours. In the last step, PEGylated squalene was synthetized by mixing 1,1′,2-tris-nor-squalene aldehyde (0.206 g, 0.54 mmol, 1 equiv.) with poly-(ethyleneglycol)-bis(3-aminopropyl) (1500 Da) (0.886 g, 0.59 mmol, 1.1 equiv.) in acetonitrile (20 mL) and magnetically stirred for 24h at room temperature (25 °C), under nitrogen atmosphere. The product was obtained in quantitative yield and used further without purification. PEGylated squalene was stored as solution in acetonitrile at 2-4 °C for further experiments. **^{1}H-NMR** (400 MHz, $CDCl_3$, TMS) δ (ppm) = 7.64 (1H, t, J=4.8, CH=N), 5.15 - 5.08 (5H, m, CH=C), 3.70 - 3.64 (140 H, m, CH_2-CH_2-O), 3.19 (2H, t, J=6.4, CH_2-NH_2), 2.53 - 2.49 (2H, m, CH_2), 2.33 - 2.30 (2H, m, CH_2), 2.09 - 1.97 (16H, m, CH_2-CH_2), 1.88 - 1.83 (3H, m, CH_3), 1.68 (3H, m, =C(CH_3)-CH_3), 1.61 (12H, m, =C(CH_3)) (Fig 7.S). **^{13}C-NMR** (101 MHz, $CDCl_3$, TMS) δ (ppm) = 161.67 (C=N), 134.91 (CH_3-C=C), 131.25 ($C(CH_3)_2$), 124.40 (CH_2-C=CH), 124,26 (CH_2-C=C), 72.57 (OCH_2-CH_2), 70.58 (O-CH_2-CH_2-O), 70.35 (O-CH_2), 70.12 (O-CH_2), 69.97 (O-CH_2), 61.71(NCH_2), 39.73 (CH_2-CH_2), 39.58 (CH_2-NH_2), 31.85 (CH_2), 28.25 (CH_2), 26.77 (CH_2), 26.66(CH_2), 26.55 (CH_2), 25.71 (CH_2), 17.69 (CH_3),16.06 (CH_3), 16.01 (CH_3).

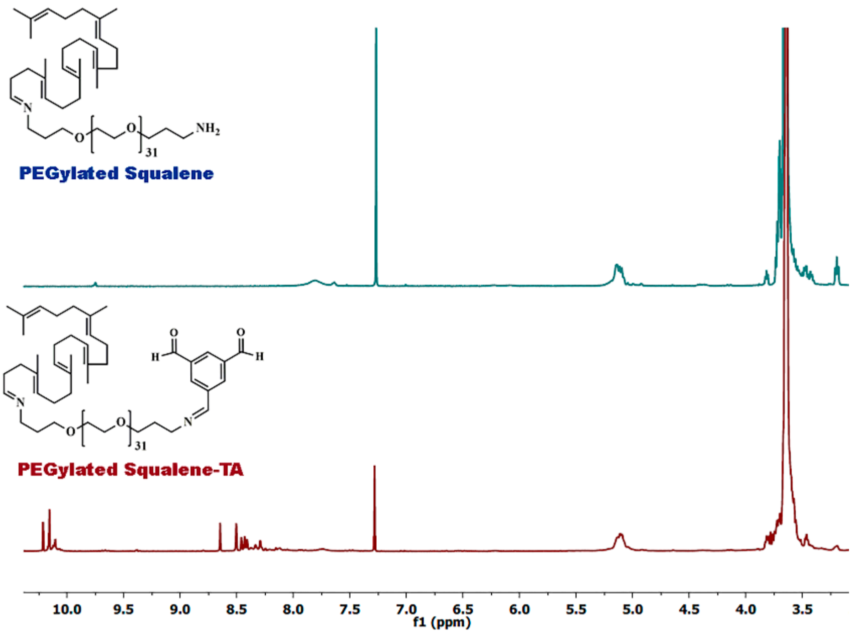

Figure S1. 1H-NMR spectra of PEGylated Squalene intermediates in CDCl3.

Table S1. Compositions for dynamic frameworks libraries synthesis.

Sample	**STEP 1**				**STEP 2**				**STEP3**			**NVs**
	SQ-PEG-NH2-TA				H2N-PEG-NH2				bPEI800			
	MW (Da)	Molar ratio (equiv)	m (mg)	n (nmol)	MW (Da)	Molar ratio (equiv)	m (mg)	n (nmol)	Molar ratio (equiv)	m (mg)	n (nmol)	Final Volume (μL)
NV1	2008.58	1	12.5	6.20	1500	0.1	0.93	0.62	1.5	7.44	9.3	3000
NV2						0.2	1.86	1.24				3000
NV3						0.3	2.79	1.86				3000
NV4						0.4	3.72	2.48				3000
NV5						0.5	4.65	3.10				3000
NV6						0.6	5.58	3.72				3000
NV7						0.7	6.51	4.34				3000
NV8						0.8	7.44	4.96				3000
NV9						0.9	8.37	5.58				3000
NV10						1	9.20	6.20				3000
NV11					2000	0.1	1.23	0.62				2247
NV12						0.2	2.47	1.24				2247
NV13						0.3	3.70	1.86				2247
NV14						0.4	4.93	2.48				2247
NV15						0.5	6.17	3.10				2247
NV16						0.6	7.40	3.72				2247
NV17						0.7	8.63	4.34				2247
NV18						0.8	9.87	4.96				2247
NV19						0.9	11.10	5.58				2247
NV20						1	12.34	6.20				2247
NV21					3000	0.1	1.85	0.62				2247
NV22						0.2	3.70	1.24				2247
NV23						0.3	5.55	1.86				2247
NV24						0.4	7.40	2.48				2247
NV25						0.5	9.25	3.10				2247
NV26						0.6	11.10	3.72				2247
NV27						0.7	12.95	4.34				2247
NV28						0.8	14.80	4.96				2247
NV29						0.9	16.65	5.58				2247
NV30						1	18.50	6.20				2247

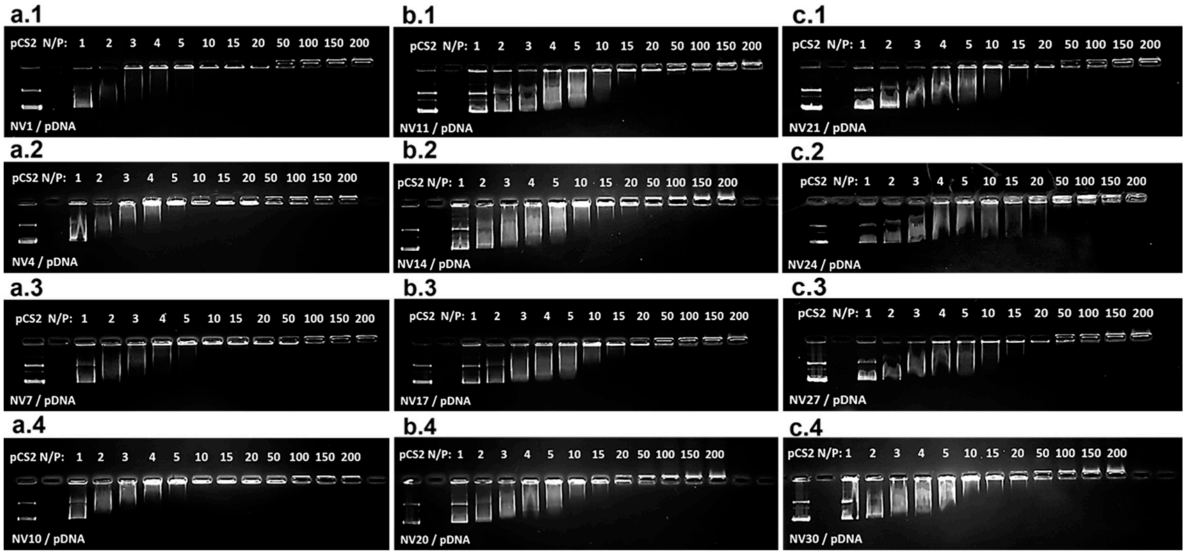

Figure S2. Electrophoretic mobility of plasmid DNA in the complexes between dynamic frameworks and pDNA at various N/P ratios. PEG-1500 Da: **a.1-a.4**, PEG-2000 Da: **b.1-b.4** and PEG-3000 Da: **c.1-c.4**.

References

1. Ceruti, M.; Balliano, G.; Viola, F.; Cattel, L.; Gerst, N.; Schuber, F. Synthesis and biological activity of azasqualenes, bis-azasqualenes and derivatives. *European Journal of Medicinal Chemistry* **1987**, *22*, 199-208, doi:https://doi.org/10.1016/0223-5234(87)90050-X.
2. Craciun, B.F.; Vasiliu, T.; Marangoci, N.; Pinteala, M.; Clima, L. PEGYLATED SQUALENE: A BIOCOMPATIBLE POLYMER AS PRECURSOR FOR DRUG DELIVERY. *Rev Roum Chim* **2018**, *63*, 8.
3. Clima, L.; Peptanariu, D.; Pinteala, M.; Salic, A.; Barboiu, M. DyNAvectors: dynamic constitutional vectors for adaptive DNA transfection. *Chem Commun* **2015**, *51*, 17529-17531, doi:10.1039/c5cc06715d.
